# Family Self-Support in Managing Down Syndrome Children: A Qualitative Study

**DOI:** 10.1155/2024/9992595

**Published:** 2024-05-23

**Authors:** Farzaneh Noroozi, Zohreh Farrar, Tayebeh Gharibi, Roqayeh Gashmard

**Affiliations:** ^1^Health Education and Health Promotion, Department of Midwifery, School of Nursing and Midwifery, Bushehr University of Medical Sciences, Bushehr, Iran; ^2^Research Committee, Faculty of Medicine, Bushehr University of Medical Sciences, Bushehr, Iran; ^3^Department of Midwifery, Nursing and Midwifery Faculty, Bushehr University of Medical Sciences, Bushehr, Iran; ^4^Department of Pediatric Nursing, Faculty of Nursing and Midwifery, Bushehr University of Medical Sciences, Bushehr, Iran

## Abstract

*Background and Aim*. Down syndrome (DS) is the most common reason for disabilities caused by genetic disorders. Due to the special nature of this disease and the special needs of children with Down syndrome, they are required to receive their families' support. Therefore, the recognition of their problems and needs and also the alternatives for resolving them and promoting their life quality are very useful. Also, since very limited qualitative studies have been conducted, it seems necessary to design a qualitative study. *Method*. This qualitative study was conducted by the content analysis method and through purposeful sampling method with the participation of 26 participants including 15 mothers, 6 fathers, 3 sisters, and 2 brothers of DS children in 2022-2023. The data were collected through semi-structured interviews. *Findings*. Using the content analysis method of Graneheim and Lundman (2004), the main theme was “Family self-supporting in protecting Down syndrome children.” The subthemes were seven including “trying to find information-support resources,” “Giving importance to child's health,” “religious beliefs of the family,” “child moral education, helping to child's relative self-support,” “developing familial support,” and “developing child's social interactions.” *Conclusion and Recommendations*. The findings of this study showed that family is the main source of fulfilling the needs of children and their life challenges through using efficient self-support methods. This study introduced family self-support methods in terms of DS children in a way that other families can also manage the problems of their children more efficiently. The present study can be used by trustees of DS to support them and their families. Considering the existence of many problems in children with Down syndrome and the involvement of families, it is suggested that policymakers and community health managers provide the basis for receiving services and social support. For example, it is possible to strengthen the screening systems in the country to diagnose the disease on time and take quick action to solve this problem. Also, by increasing the health insurance coverage and fair distribution of the support resources needed by these people, it promoted the quality of life for them and their families. Also, health policymakers in Iran can take action to increase life expectancy and reduce deaths caused by DS by improving the equitable distribution of health resources and services. Also, public policies should enhance supportive intermediation for prevention and life quality promotion and also decrease health challenges. They are also supposed to lessen the costs of health care. Furthermore, to support social organizations, health service providers and researchers should consider the development of intermediations for the health enhancing and life quality promoting of DS children.

## 1. Introduction

DS or Trisomy 21 is the most prevalent reason for cognitive disabilities caused by genetic disorders [1, 2]. By increasing the age of marriage, the rate of this disease prevalence has developed [3, 4]. In the world, out of every 800 live births, one child is born with Down syndrome. In the U.S., 5000 DS children are born and over 200000 DS live [2]. The prevalence of DS and its related mortality in Iran in 2019 were about 29.31 and 0.34 per 100,000 people, respectively, which is lower than in the Eastern Mediterranean region. The African region and low-income countries had the highest rate of mortality caused by DS, and the European region and high-income countries had the highest prevalence of DS. At the national level, Romania and Burkina Faso had the lowest (0.02 per 100,000 people) and the highest (1.26 per 100,000 people) mortality caused by DS, and also Taiwan and Brunei had the lowest (8.32 per 100,000 people) and the highest (97 per 100,000 people) prevalence of DS among the countries studied [5].

Studies have shown that these children suffer from different problems such as motor-neural growth delay, diabetes type 1, obesity, and also cardiovascular diseases [1, 6, 7]. These disorders cause a decrease in their quality of life and also increase the need to receive lifelong care services [3]. Children with DS show lower levels of the overall QoL than children without DS though, with varying levels across the QoL domains [8].

Also, they face various challenges such as social isolation, the limited time to manage their personal affairs, and other stressful factors. A few seminal studies have demonstrated that those with a disability may be at a higher risk of being excluded when compared to TD peers [9]. It is shown that lack of social support, and the widespread stigmatization confronting children with DS and their families, hinder the development of positive and empowering adjustments that would best serve the child's and the family's interests [10]. Parenting a child with Down syndrome (DS) is associated with demands on parents that make combining care with other responsibilities challenging. These demands generally concern the initial adaptation to the diagnosis of the child, the caregiving demands, the coordination of services, financial consequences, and societal attitudes. Consequently, parents of children with DS have been found to show higher levels of stress than parents of typically developing children [11].

On being told their newborn baby has an impairment, parents tend to react with a mixture of shock and disbelief, followed by denial. As the reality becomes undeniable, feelings of guilt, fear of the reactions of others, and uncertainty regarding the future emerge. Torn between powerful and conflicting emotions, parents live through a stressful period in which coping depends on support from one another, extended families, friends, support groups, and skilled professionals. The needs of children with different impairments differ, as do the practical and emotional challenges that families face in providing for them [10].

When a baby with DS is born, parents experience many conflicting emotions and express feelings of ambiguity or incompetence. Because many children with DS experience complex health problems and social stigma, family members might have continuous stress. Parent-child relationships are disturbed; mother-child attachment is delayed, and so is the growth and development of the child, which can lead to disturbed family functioning [8].

Due to advances in the medical treatment of people with DS, their life expectancy has increased markedly in recent decades. This change in life expectancy requires researchers to think about the social support networks of people with DS from a life course perspective. Because of the learning disabilities, health issues, and an increasing risk of age-related diseases that individuals with DS face, they require robust social support networks throughout their lives to prevent them from becoming isolated, to help minimize the adverse health effects of the condition, and to promote “aging in place” to avoid long-term care in nursing facilities. People who have no relationships with others are socially isolated, as opposed to socially integrated individuals, who are generally involved in formal and informal relationships. Social networks provide individuals with important resources, such as information, access to jobs, improved health, and overall support [12].

Additionally, these children impose more financial and emotional costs on the family than their healthy children [13]. Little attention to the child's emotional well-being was observed, with none considering family appraisal of the child's emotional well-being. The relationship between family variables and child QoL was rarely the primary focus of the study [8].

The relationship between people with mental and physical disabilities, their families, and social relationships with others is a very important issue. Besides, the family has a very important role in improving or reducing the communication skills of children with disabilities, which can limit, maintain, or increase them [7]. The disability of children has a considerable impact on family function and makes them involved in extra responsibilities, high concern concerning about children's care needs, their educational and medically necessary services, high cost of children's vital services, ambiguous future, social isolation, loss of social opportunities, overabsenteeism in the workplace, and financial, physical, and emotional challenges [14–16]. The results of the study showed that the social networks of the caregivers interviewed were predominantly small, not very dense, and featured strong relationships with family and friends. From an analysis of the interviews, three categories were identified: emotional support from the family, emotional and financial support from friends, and dissatisfaction with the health service [17]. In another study, families described challenges to achieving appropriate care coordination and stress associated with healthcare navigation. Some facilitators to screening described included insurance, personal connections, primary care, allied health professionals, and family support groups, and some barriers included ineffective care coordination, insurance navigation, and healthcare provider shortages [18].

The findings in terms of treatment and rehabilitation of children with special needs emphasize the critical role of parents as the main carers of children's supporters. These interventions have several goals: they are supposed to prevent child abuse and neglect, increase their knowledge and skills, improve their skills in using equipment and facilities, and strengthen their ability to adapt to the disease [19].

Family-centered care requires looking at the family as the center and the core of care, which despite its many advantages, this type of care has received less attention and unfortunately has been neglected in Iran [20]. The presence of a child with mental and physical disabilities tremendously affects family members and can lead to long-term consequences of hidden and apparent disturbing changes affecting their life in different aspects [21]. The results of a study revealed that the age of the child, developmental level of the child, parental depression, and stress and strain were negatively related to family adaptation, whereas the health of parents, family cohesiveness, flexibility, communication skills, supportive family/relatives, and quality of community service were positively related [22]. Also, the results of a study revealed that the building approach includes strategies that rely on family members and close friends for building a support network for the person with DS. The connecting approach includes strategies that connect the person with DS to external and often professional resources and services [12].

Los pinas et al. (2017) showed that families having disabled children suffer from some problems including an imbalance in the family system, a lack of awareness regarding different aspects of health and education, negative feelings, and the need for adaptation. However, they decided to focus on developmental skills such as communication skills, self-esteem, being happy, and having more flexibility. These potential skills were reinforced in the family and led to improved quality of life and caused them to get closer to the community [23]. In another study, that sample was divided into three different groups: (1) subjects with TD children, (2) subjects with DS children, and (3) subjects without children, and the results showed that people with Down syndrome are considered less competent in social skills. There are differences in beliefs between the three groups: parents with DS children have more positive beliefs about all skills, especially regarding socialization [24]. Children with DS may be at an increased risk of impaired social functioning compared with normal controls. Children with DS generally have poorer social abilities compared to nonverbal mental age-matched controls, and they also have more social problems than chronologically age-matched typically developing controls and those with similar nonverbal mental age [25].

Bray et al. findings indicated that peers supporting the family have a positive impact on resolving the psychological problems of parents and increasing their abilities to adapt to their disabled child. The parents contributing to this project were guided by their peers and fought all the time for survival and a better life. They also paid attention to the barriers that might stand in their way and changed to adapt more in life. Empowering parents by sharing their feelings, concerns, and anxieties with parents who have already traveled this path was considered an important part of this support project. Also, parents' development and their social relations increased and became more complete. This project assisted parents in changing their viewpoints from a failed person to a dominant person. They learned that exposure, acceptance, and fighting were the main elements of development. They should increase their relationships with others and pass from their previous condition quickly [26]. Also, mothers held aspirations for their children's future that included autonomy and independence; however, their capacity to promote autonomy was sometimes constrained by a range of child and family factors, as well as by their focus on developing skills for independent functioning [27].

Since the birth of a disabled child leads to family shock and results in their depression and being more vulnerable, they need to receive support from others. However, their relationships decrease leading to worsened conditions, and social support is limited due to loneliness and frustration. These, finally, cause mental, physical, and communicative damage to the family [28].

For instance, based on the reports released by Bushehr province's Welfare Organization, 308 people with DS are living in Bushehr province now. After the birth of a DS child, different problems occurred for the patient and his/her family. Their problems consist of personal, social, cultural, and familial aspects.

Also, DS suffers from various problems including cardiac disease, hearing and thyroid problems, and speech and motor defects. Therefore, they require family and other social support [1]. In addition, the costs related to the follow-up of treatment, education, rehabilitation, counseling, etc., are very high, which imposes a heavy economic burden on the family and takes a lot of energy from them.

These families need to gain enough support due to various problems and provide care services to their patients [28]. Multidimensional problems (physical, mental, social, etc.) of DS children affect his/her life aspects and their family's life [14]. These children have special needs related to their disease and growth and development challenges in addition to their public health problems [20].

The problems associated with the learning process in these children cause them to spend an ample of energy by family in terms of educating social skills for acquiring the abilities of communication [29]. Owing to insufficient sources of financial and social support for DS children's families, the importance of investigating the experiences of these families, and having done limited studies related to this issue, the researcher of the present survey decided to conduct research concerning family self-support for managing DS children.

## 2. Methods

This project was initially affirmed by the Research Council and Ethical Committee of the Research Vice-chancellor of Bushehr University of Medical Sciences. This investigation was conducted by a qualitative content analysis.

The participants of the present study included DS child's family members (father, mother, sister, and brother) who registered with the Welfare Organization of Bushehr province and had medical records. In these centers, medical data of DS children were extracted.

Many Iranian families use the services provided by the Welfare Organization for DS children. However, the number of these centers is limited in the country. They provide some services such as speech therapy, painting classes, and teaching life skills such as interaction with others, personal hygiene, and training in reading and writing.

Semi-governmental professional education institutes monitored by the Welfare Organization provide several supportive educational services such as teaching, tailoring, and carpentry for people over 14 years old. Their costs are paid by the family and the government. In total, 26 families of DS children family members participated in this study. Meanwhile, 3 of the families refused to be interviewed. Some information presented by the DS children's siblings led us to invite them to contribute to the present study.

Classic content analysis was used in this study. Content analysis is a method that is used to produce reliable and repeatable results from related data, and its purpose is to produce new knowledge and perspective that represents reality and a guide for action [30].

Qualitative research is designed to discover the complex phenomena faced by clinical experts, healthcare providers, policymakers, and consumers of the healthcare system [31]. Content analysis is a systematic coding and categorization approach that is used to discover a large amount of textual information to identify trends and patterns of relationships [32].

This approach is the most common method used in qualitative research and aims to provide key points in participants' responses. This approach is useful for answering questions about issues important to certain groups of respondents or for identifying common responses [33].

When researchers intend to determine the structure and social factors that cause the formation and impact on behaviors, using qualitative studies will be a suitable method [34]. In this study, a qualitative research method with a conventional content analysis approach was used. In this approach, classes are developed inductively using a textual or verbal interpretation of data [35]. Content analysis is widely used in qualitative research to extract meanings. Approaches and different methods of content analysis have been used in nursing research, and their identification is a great help in choosing the right method for research.

A purposeful sampling method was used in this study, and it was conducted from November 2022 to March 2023. It was done in rehabilitative centers managed by the Welfare Organization of Bushehr City or the houses of families having DS children. The participants of this study were 26 people, including 15 mothers, 6 fathers, 3 sisters, and 2 brothers of Down syndrome children. The main participants of this research were their mothers because of their key role in the management of children with Down syndrome. The inclusion criteria for the participants included interest in participating in the study, having living experience with Down syndrome children, and the ability to communicate and express their feelings and experiences. Also, the inclusion criteria for family members included continuous communication with the child and interest in participating in the study. To enter this study, the age range of children with Down syndrome was considered from birth to 18 years of age. First, targeted sampling based on the above criteria was used to select the participants. In targeted sampling, 15 mothers were included in the study. To select the participants, the researcher referred to the rehabilitation centers under the supervision of the welfare organization-identified qualified people and invited them to the rehabilitation centers or any environment where they felt more comfortable. Then, more participants were selected in the theoretical sampling process and included in the study so that after the initial analysis of the data from the first few interviews, all the resulting concepts and propositions were followed up in theoretical sampling. By using theoretical sampling, the researcher took advantage of the people who maximized the opportunity to develop the features and dimensions of the concepts and discover the differences and relationships between the concepts to collect data. The criterion for determining the sample size in this study was to reach theoretical saturation, which means that no new concept appears, the evolution of concepts in terms of their characteristics and the variety of their dimensions.

Data collection was done by a semi-structured interview with the participation of 26 DS family members (15 mothers, 6 fathers, 3 sisters, and 2 brothers) with purposeful sampling at first and then an opinion to achieve the greatest diversity and data saturation. The age of the children was 4–18 years (10.6 ± 1.35). 12 of the children were girls and 14 were boys. Parents' age was between 37 and 54 years (44.4 ± 3.26).

The education level of parents was a high school diploma (9 cases, 43%) and higher than a diploma (12 cases, 57%). Additionally, 3 fathers and 3 mothers were employed. All families had just one DS child. The criterion for determining sample size, in the present study, was the time at which saturation happened. Consent forms were completed before the interviews accompanied by a sufficient description given by the interviewer. Data collection was done by using a definite question, and then it was continued through a semistructured style. All interviews were recorded. Interview questions were started with this test question: “When you found that your child is a DS, what did you do? After the participants responded to this question, the next question was asked: “What do you or did you face with this issue?” Based on the responses, some questions were asked to receive deeper answers and attain further information.

For instance, “Can you express any example of your daily life experience of living with your child?” Exploring and following up questions were also asked based on participants' responses, such as please describe more or “What does it mean?” Interviews lasted between 40 to 55 minutes. Data analysis was done during data collection based on the stages suggested by Graneheim and Lundman [36].

The results of the Masoudi et al. study showed that Granheim and Lundman's inductive approach was used the most in 135 articles (43.97%). In this method, to perform content analysis, codes are created for meaning units. The meaning unit can be a sentence, paragraph, or whole text. The purpose of coding is summarization and condensing of meaning units. A total of a few codes lead to the creation of the category. A category is a group of content that is conceptually similar, and refers to a clear topic. A category can have subcategories. Categories are homogenous from within and heterogeneous compared to other categories. Each category should be comprehensive and unique. According to Granheim and Lundman, no similar information should be shared between the two categories, and of course, no information related to the purpose of the study should be included. Of course, no information related to the purpose of the study should be removed due to not having the appropriate category, and finally, the theme is formed. Levels of abstraction increase from code to content. The inductive approach and the Graneheim and Lundman method are more interesting to Iranian researchers. In the inductive approach, researchers seek to find similarities and differences in the text, which are explained in separate categories and themes. The researcher moves from data to theoretical understanding or from specificity and objectivity to generality and subjectivity. One of the possible reasons for the attention of this method by researchers is its simplicity. The four stages of doing this method in brief include the following: 1. writing and rereading the text several times, 2 and 3. identifying meaning units, classifying, and considering a suitable label for each of the categories, 4. sorting the subcategories based on comparing the similarities and differences between them and finally considering a suitable general label that includes the concept of categories [37].

In Granheim and Lundman's method, the levels of abstraction from low to high are meaning unit, condensation of meaning unit, code, subcategory, category, and finally theme [36].

Typing of texts was done word by word without data processing software. To get a general perception of the data, the transcribed interviews were read several times by the researcher before coding. After reviewing the transcribed interviews and determining the main sentences and concepts in each line or paragraph, semantic units were assigned.

Summarized semantic units, using names of the main codes, and by connecting similar concepts, a new phenomenon was obtained. Then, subthemes and themes were extracted. Finally, based on the concepts in the subthemes, the relevant theme was obtained. For example, the theme “religious beliefs of the family” is produced from several subthemes such as belief in divine providence, worship to obtain peace, and caring as worship. The validity of data was obtained through long-term work with participants, member checks, and peer checks. For member check, the rewritten interviews along with the extracted codes were given to three randomly selected participants whose opinions agreed with what the researcher evaluated.

Peer check was done by giving transcribed interviews and extracted categories and codes to research team members. In addition to that, participants with maximum diversity in terms of life experience with a Down syndrome child and from different demographic groups and children with different degrees of Down syndrome were used, and the increase in the number of interviews with the participants was done due to the increase in the transferability of the findings. Also, during data analysis, external auditing and recoding methods were used to increase reliability [36].

### 2.1. Ethical Considerations

This article is a part of a study entitled explaining the process of managing a Down syndrome child in the family, approved by the research council and ethics committee of Bushehr University of Medical Sciences, which was approved by the ethics code IR.BPUMS.REC.1401.135. At the beginning and before the participation of each participant, the researcher explained the objectives of the research to them and made them aware of the voluntary nature of participating in the research, the confidentiality of personal information, and the possibility of withdrawing from the study at any time.

The researcher assured them that if they left the study, all their conducted interviews and texts would have been returned to them and papers including their words would have been kept safe and then destroyed after releasing the results of the study. The time and place of the interview were determined by the participants.

### 2.2. Findings

Finally, 400 conceptual codes of data were divided into seven themes. Every theme had 2–5 subthemes as follows.

### 2.3. Family Self-Support in Managing DS Children

This theme refers to family self-support in supporting DS children based on their capabilities for this goal which includes the methods that the family uses to compensate for the child's deficits and limitations, as well as reinforcing and empowering the DS child. Subthemes consisted of “trying to find supportive-informative sources,” “giving importance to the health of the child,” “family religious beliefs,” “child moral education,” “child's relative self-support,” “developing familial support,” and “social interaction development.”

#### 2.3.1. Trying to Find Information-Support Resources

Experiences of DS children's parents showed that they tried to gain supportive-informative sources for meeting these children's special needs. This theme included three subthemes consisting of “trying to be familiar more with a child's supportive needs,” information acquisition for meeting a child's supportive needs,” and also “gradual development of information-support resources.”

The first line of parents' information-supportive source was the health team including physicians, nurses, health staff, consultants, and social workers. A mother of a DS child said in this regard:

*“From the beginning, I was trying to find necessary information. I asked the health staff about my child's condition and growth and this point that where I should go. They told me that these children have slower growth than other children and they should be visited by a pediatrician and I did so”*. (Participant 3).

However, the majority of families did not limit their information-supportive sources to health team members. They tried to get familiar with the families having children with DS to obtain information. In addition, they kept in touch with Welfare Organization centers for developing their supportive-informative sources.

One mother said in this regard:

*“When my child got older, I got familiar with a woman suggesting me to take my child to a rehabilitation center. She said that they trained him, they did speech therapy and I got encouraged and registered my child in this center”*. (Participant6).

Another mother noted that she participated in rehabilitation, speech, and occupational therapy classes to help her child and also increase her information and skills. Gradually, supportive-informative sources developed in some families, and through the Internet and virtual space, they also developed their information to meet the needs of children. One of the mothers said

*I am so interested in gaining information about DS so that I can help my child, I searched on the Internet by myself to find educational subjects and study*”. (Participant 10).

Also, another mother said

*“I read the books to find more information about Down syndrome so that I could help him more”* (Participant 4).

#### 2.3.2. Giving Importance to Child's Health

With the development of parents' information and their constant exposure to the needs of children with Down syndrome, parents gradually realized that these children, due to their special physical conditions, are more prone to suffering from many disorders such as visual, hearing, and speech deficits, mobility limitations, and other diseases than typical development children. In addition, they have many problems such as heart and thyroid disorders, etc., so they need more care and if they are neglected, it can enhance the physical and mental stresses of the family. Therefore, the parents of these children paid special attention to ensuring the health of the children and preventing their physical problems. This theme has two subthemes including the treatment of physical problems or disorders and attention to disease prevention.

Some participants emphasized pursuing several measures such as annual child check-ups, vaccinations, personal hygiene, and nutrition in a way that they can provide a healthier life for these children.

One of the mothers said this:

*“If the children are not taken care of and so they get ill, we will get embarrassed and I got hurt more*”. (Participant 8).

Another mother also stated her belief about taking care of children:

*“Because he is disabled, I have to take care of him. I look after him so much, and for example, I look after his nutrition. I did all his vaccinations and health care completely. He is under pediatric supervision. I take him for speech and occupational therapy, though it is too far from our house and it is difficult for us and we cannot afford it, but when he is physically ok, I and my family feel better”.*(Participant 1).

A mother also noted that

*“My child has different physical problems such as tear duct obstruction, cataract, hyperopia, diabetes, hyperlipidemia and she needs various kinds of surgeries”*.

She emphasized that

*“These children require more health care, otherwise their deteriorated physical problems will lead to further pressure on the family”.*

She also pointed to children's vulnerability to obesity:

*“These children are prone to diabetes and obesity, so I put my child on a diet for losing weight. I take her for a check-up every year because they are also prone to thyroid problems*”. (Participant 10).

Another mother also declared

*“I'm very careful about the symptoms of illness in my child and as soon as I feel that something is wrong with him, I act very quickly and I pay a lot of attention to prevent worse complications in my child”.* (Participant 2)

#### 2.3.3. Religious Beliefs of the Family

The experiences of the participants in this study showed that the presence of religious beliefs in the family played a major role in accepting a child with Down syndrome. This theme has three subthemes: “belief in divine providence,” “worship to obtain peace,” and “caring as worship.” The participants considered the birth of a Down syndrome child as “providence,” “destiny,” or a divine test and they believed that accepting divine providence and trying to succeed in the divine test, while strengthening the foundations of the family and expanding peace, lead to the opening of divine doors to he turns to them.

One of the mothers stated her belief in God's will as follows:

*“All the time, I feel that God is with me, God loved me and gave me this child. He will reward me in this world and the next. This causes me to accept this situation better and can keep up with it*” (Participant 5).

Another mother said

*“These children are like angels, I feel to be closer to God and get more calmness by looking after him”.* (Participant 3).

Alongside these deep beliefs, doing some rituals such as prayer and reading the Quran reduces tiredness and increase calmness in mothers.

One of the mothers stated this

*“Whenever I feel tired and under pressure, I say a prayer for my family, child, and all patients and I ask for health and well-being for them”*. (Participant 7).

A sister pointed to her father's advice on spiritual reward and taking care of her brother:

*“My father always tells us taking care of your brother is so valuable for God. He reads the Quran whenever he feels pressure, then he feels calmness and prays for all*”. (Participant 19).

These beliefs in family cause calmness and satisfaction for all the problems related to taking care of DS children for some participants which can be gained only through acts of worship.

The other sister said the following in this regard:

*“These children are innocent, I feel to be beside one of God's angels. Whenever I do something for him, I have a spiritual emotion. It is very enjoyable. Getting close to an innocent guy gives calmness to anybody”*. (Participant 24).

A father also declared

*“Perhaps some people think taking care of these children is so difficult, but I have felt that God pays attention to us due to this child. So, I regard giving services to him as a way of worshiping God”*. (Participant 16)

#### 2.3.4. Child's Moral Training

One of the child's needs which the family tried to fulfill was to do moral training in a way that whenever the child's behavior and words were rude, the family tried to modify these inappropriate behaviors or words by kindness and affection and through supportive and noninvasive methods. The participants tried to apply training measures to the child in such a way that they could strengthen the correct and appropriate behavior and speech in the child. Families' experiences in this domain included three subthemes as follows: “teaching moral principles to the child,” “regarding moral principles by family members,” and “appropriate behavior model of parents for family members and relatives.”

The parents taught moral principles to the child by encouraging other children to take appropriate behaviors, lack of reinforcing the child's inappropriate and also abstain from doing appropriate acts in his/her presence. One mother said in this regard:

*“We teach him to respect the elders, say hello to them and we emphasize to him not to wrangle with anyone else, not to tell any bad word or insult no one. Whenever he does something good, we encourage him by appreciating words and inga reward. For example, we make an encouragement card for him which marks a star on it for every good behavior which he does and rewarding a way that this good job would be reinforced*” (Participant 9).

One father stated as follows in respect of regarding moral issues:

*We do not do any wrong action or even childing in presence of this child, even the movies which we watch, are controlled more when he is in so that they have no aggressive or appropriate behaviors”* (Participant 19). Another father also said:

*“I try to show attractive children's cartoon CDs that have educational and moral content for my child”*. (Participant 21)

Struggling for good role modeling is one of the methods that participants utilized for teaching children morally. They accompanied the child in some social interactions in addition to trying to provide behavioral appropriate models for their child and to teach other individuals how to interact with this child.

One mother said about this

*“I and my couple try to be a good role model for family members and show them the correct way of treating DS children through our behaviors. For example, we never disrespect him, empathize with him all the time and treat him such a typical development guy, not a patient”.* (Participant 7).

Another mother stated about their role-modeling of appropriate behaviors for DS children:

*“I and my couple and my other child, play with him in a group for showing him correct behaviors. Routinely and in our plays, we ignore his incorrect behaviors and speeches so that their repetition would be less. Instead, we practice correct behavior or speech in a way that he learns what correct behavior is, for example, how to speak and how he behaves in similar situations. We to teach him to say hello, introduce himself to others, or invite someone else as a playmate”.* (Participant 10).

#### 2.3.5. Helping to Child's Relative Self-Support

In this study, the experiences of participants showed that the constant struggle to acquire information with respect to meeting a child's needs *s* led to gradual failure in achieving a child's relative self-support. This thrive represents an ongoing effort of the family to empower children, making them independent and relatively self-supported.

This theme has five subthemes: personal hygiene teaching, vocational training, entrusting personal work, and some other matters to him/her and giving him/her the power to choose and invest for his/her future.

One father stated about this issue

*“We always try to find a way to help our child and teach him in a way that he can stand on his own feet*”. (Participant 20).

Another mother declared

*“We take him his own personal affairs like personal hygiene, clothing, feeding etc. We worked on these matters so much until he got independent. We teach him to help us in household affairs, like folding clothes, though he cannot do them well or give him a broom to sweep*”(Participant 5).

One of the fathers talked about giving power to DS's child:

*“We sometimes ask him to tell his idea, especially the jobs of his own, like what clothes he likes to wear, or what kind of hobby he likes to do or where he likes to go”.* (Participant 15).

Families make constant efforts to seek help from consultants, social workers, specialists, rehabilitation centers, and also speech *y* therapists. Although the services of these centers are limited and less efficient, this did not lead to disappointment in the family. One of the fathers stated this

*“Though occupational therapy done by the Welfare Organization was just one time per week and was not so effective, we took my child to private centers. Every progress was important for us. Even we accompanied with the child in the educational sessions so that we could do the practices in-house that would lead to better results”.*(Participant 21).

Also, one mother said in this regard:

*“I took my child to a carpentry workshop so that he can learn to work and be a little independent in the future”*. (Participant 6)

One mother emphasized her effort to make her child independent and stated as follows:

*“I took my daughter to private vocational training centers for a while and she learned sewing to some extent. Now she works in a tailor shop”.*(Participant 7).

Another mother expressed concern regarding personal hygiene teaching:

*“At the age of four, I regularly spent time with her day and night for a whole month, asking her and taking her to the W.C. I didn't praise her anymore, I wanted to show her the way so that she knows where she should go. At first, she said “she can't”. I repeated so much that she would learn. Of course, he would wet himself the first, the carpet was dirty and my work would be doubled, but I worked with him day and night for a whole week until he finally learned where to go to the W.C. Of course, it took several months for him to learn completely and not to wet himself anymore”* (Participant 10).

Also regarding investing for the child's future, one of the fathers said

*“I have opened a savings account in the bank for my child so that he will face fewer problems with living expenses in the future”* (Participant 17).

#### 2.3.6. Developing Familial Support

In this study, the family members of children with Down syndrome tried to develop the child's support in the family so that by sharing all the family members, the burden of care is distributed among the family members, and the extra pressure on one of the parents or the main caregiver is reduced. This theme also has two subthemes, namely, strengthening the support and participation in the family” and “following up to receive support services”.

The subtheme “strengthening the support and participation in the family” indicated the attention of family members to the necessity of supporting the main caregiver. They recognized that they should show empathy, emotional support, and appreciation for decreasing the burnout of parents so that they can do their hard-caring jobs. The alternatives that the participants used included empathy development among family members, practical participation, reciprocal and emotional support done by the couple, and encouraging siblings to contribute to the DS child's affairs.

One father stated his viewpoints about how his family members accepted DS's child and also he emphasized the importance of increasing empathy among families to make support DS's child he continued:

*“Our family relations are very good, we support and help each other, especially, we love this child so much, we regard his needs and accept him”. (*Participant 17).

With respect to family participation and emotional support from each other, a mother said

*“My husband knows how much I have worked for this child, he empathizes with me so much and supports me emotionally*”. (Participant 4).

Another mother said *in this regard*

*“My husband helps me in the child's affairs, for example, he is responsible for my son showering and cleaning when he is at home”. (*Participant 2).

Also, a mother stated how she could persuade her children to participate in care affairs by describing their brother's condition:

*“I always describe his mental and physical problems to other my children and remind them that he needs more care, they help me as far as they can. My daughter often teaches her brother his school homework and plays with him to make him happy*”. (Participant 6).

A brother also said

*“Every of my family members tries to help him in his job so that the pressure put on our parents would be decreased and they have more comfort and rest”*. (Participant 23).

The subtheme “following of receiving supportive services” represents parents' struggle and family members' struggle for having access to social support services and using them to help a DS child and empower him/her. Utilizing some services such as speech and occupational therapy can reduce the pressure he has from his parents in addition to developing his communicative and practical abilities. Parents seek for centers where they can provide services for children to help them and compensate for their limitations. By providing these services, both children and their families felt more satisfaction and their life quality increased.

One of the mothers stated about rehabilitative affairs as follows:

*“I bring him two times per week for occupational and speech therapy. In the occupational therapy center, he works out and draws pictures. As I bring him for these services, his condition gets better”.* (Participant 1).

Another mother expressed her experiences about providing education for DS's child:

*“About providing education and the time of his school beginning, I was asking so many questions till he got six years old and I was told to take him to school. First of all, they took him a test and told me that he learned nothing. I persisted so much that they let him be trained. Finally, they told us we give him another chance, and if he learns something, we will continue and if not, don't bring him anymore. I was taking him regularly to school and we were training him at home until he learned”*. (Participant 6).

One father also stated

*I maintain my relationship with the welfare and rehabilitation organization to get services for my child and I go there regularly to use them if there are services for my child”.* (Participant 17)

#### 2.3.7. Developing Child's Social Interactions

In a recent study, families tried to make connections between children and the community, friends, and acquaintances. They also tried to prevent them from being isolated and facilitated their social relationships with other individuals.

In this study, the families tried to provide the appropriate situations for the child to communicate with others to avoid isolating him, and they tried to enable the child to enter the community and communicate with others. This subtheme also included the family's effort in teaching how to communicate with others, such as interacting with the children in the neighborhood and school and increasing the ability to make friends and communicate with the teacher, as well as efforts to prevent his social isolation.

In a recent study, families also tried to make connections between DS children and others and assisted them in making friends. This theme has three subthemes including teaching social interactions to children, prevention of social isolation, and encouraging children to be independent in getting in touch with others. A father stated his experiences concerning encouraging his child to be in the community:

*“We try to grow-up him in a way that he would be like a typical development child, for example, he goes out, splays with other children, I take him out and encourage him and stay with him and ask other children to play with him. I gather other children and play football and put my son as the gatekeeper and say to them that this child is your friend too, play with him”(*Participant 18).

One of the mothers also explained

*“In the beginning, I took him with me to the group of typical development children, and by explaining that he was harmless and kind, I asked the children to be friends with him and not bother him.*” (Participant 15).

Another mother also stated her insistence on the child's presence in her family gathering:

*“Even though my family did not agree, on the day of my daughter's proposal, my Ds child will be present, maybe he will make the suitors run away. But I didn't accept and said that if they were going to like my daughter, they should like me under this condition. In the marriage ceremony, my Ds child had a tray in his hand and was serving the guests. Now my son-in-law won't go out without my DS child. He says that he should too let's go together”* (Participant 2).

Another father also said

*“I take my child to the park every day so that she can play and teach her to greet children and make friends”.* (Participant 16)

## 3. Discussion

This study was conducted to explore the experiences of families having a DS child in order to study the ways for achieving self-support with respect to protecting DS children. Although the birth of DS children leads to parents' and families' depression, anger, shock, and grief [38, 39], the studied families in recent research passed this critical stage, accepting conditions and taking different ways of self-supporting and helping them. In line with this study, Skotko et al. (2016) reported that positive themes tend to dominate within modern-day families who have members with DS [40].

“Trying to find information-support resources” is known as the main and most remarkable way for self-support and helps the family to become familiar with the child's needs, various alternatives to fulfill these needs, and developing self-supportive ways in addition to attaining calmness. The primary informative-supportive source of parents is medical staff including physicians and nurses [41]. For example, pediatricians play an important role in the care of children and adolescents with Down syndrome and their families. Awareness of the issues important to affected children, adolescents, and their caregivers can make a great difference in outcomes across the lifespan [42]. It is necessary that health personnel, particularly nurses, address the subject in order to discover the relationships and how they are established, so as to strengthen bonds and carry out interventions to improve the quality of life for caregivers of children with Down syndrome [17].

Although the primary informative-supportive source of parents is medical staff including physicians and nurses, Tallon et al.'s study (2017) showed that medical staff have focused on disease and therapies as before, instead of focusing on family-centered care. However, they should have more comprehensive viewpoints in terms of social and psychological factors determining health for families having children with chronic diseases [43].

In this study, in addition to medical staff, the families developed their information-support resources for other resources such as acquaintances, online sources, and social networks. Developing information-supportive resources for parents helped them accept their children, fulfill their needs, and provide any facilities and circumstances in which DS children could grow up. Rassafiani et al. (2011) mentioned the necessity of providing information for families having DS children and reported that these families wanted to hold more seminars in order to become familiar with this disease and receive educational books and materials through which the parents could learn how to behave with these children [44].

A study conducted in Taiwan also reported that parents of DS children were continuously trained by health educators. These parents tried to learn how to stimulate their children's mental processes. They also kept in touch with healthcare services and specialists responsible for allocating social supportive sources [45].

Their familiarity with supportive sources causes parents and families to gain more skills and adaptability for addressing their problems, modifying their life plans, and supporting their children; their survival power increases, and finally, they can grow up [41].

The study of families with disabled members has shown that family information development helps these families focus on their developmental skills such as communication, self-confidence, being happy, and adaptability, instead of focusing on problems. Therefore, regaining balance in a family brings about not only high life quality, but also, it leads them to be closer to society [23].

The subtheme “following of receiving supportive services” shows the endeavor of the family to attain and use social support and services in order to develop the child's mental and physical condition and also assist in improving his/her capabilities. In a recent study, supportive services such as occupational therapy, speech therapy, and skill learning were used to help DS children and their families. Having accessibility to supportive services can foster children's empowerment and optimism about the future and also decrease the stresses that families undergo.

Paster et al. found that seeking social support was the main adjustment method for families with disabled children [46]. Christian also showed that mothers with no social support had a lower chance of committing health promotion behaviors. In addition, children living in poor families with weak financial support or those suffering from chronic or acute diseases could affect their family members' life quality more negatively [47].

Samadi et al. demonstrated that social support can increase the emotional health of parents having disabled children, though it may not be impacted by family function seriously [48]. Also, the accessibility to social support can affect the perception of financial burnout and family adaptability [49].

Bryant et al. reported that peer support (receiving guidance from similar families) can lead to survival, adjustment, and more efficient fighting of families with disabled children. It also improves parents' capabilities and their social communications and also changes their situation from a lost person to a capable and ready-for-fight individual [50].

Healthcare providers should be aware of their responsibilities for monitoring the welfare of caregivers of disabled children. Specifically, health specialists, society, and families have to regard ideal conditions for assessing the health condition of children caregivers. Since health staff keep in touch with children and their caregivers during ongoing interactions, they can consult them with respect to protecting health, assessing health needs, and also providing preventive care and if needed, referring process. In this regard, Leedom also declared that one of the barriers to optimal healthcare and medical screenings for children with Down syndrome was healthcare provider shortages [51].

Additionally, psychological challenges such as parent stress have been a topic of debate among the caregivers of children with any disease. Due to the harmful effects of psychological issues on caregivers of disabled children, the importance of intervention for these people is obvious, especially on the consequences of their physical and mental health [52].

The theme of “giving importance to child's health” not only showed the inevitable attention of the participants and especially the parents to the physical problems and disorders of the child but also was considered a chosen and conscious solution to reduce the physical and mental pressure on the family. In fact, the parents gradually realized that these children's susceptibility to various diseases and disorders can provide the basis for increasing the physical, mental, and economic burden on the family.

Therefore, regarding the health needs of children can not only keep them healthier but also diminish the mental, physical, and economic problems caused by this disease.

As a result, they spent hard efforts on the treatment and prevention of deteriorating disorders that these children face with. In line with this study, Caldwell et al. [18] reported that the following themes were identified as facilitators of healthy habit formation: [1] on the move and [2] sound sleep. The barriers also included [1] co-occurring conditions and [2] eating behaviors. Ease of access to parks, walking trails, specialists, and opportunities to promoting health were valued. The level of social support from friends, family, and other parents of young children with Down syndrome also was a key contributor to building healthy manners [18].

The experiences of the studied participants showed that while the struggle to find information-supportive sources guided parents and family members to provide therapeutic and care-related needs, “religious belief” such as belief in divine providence, belief in the innocence of DS children, and saying prayer and worship “caused the DS child to accept DS and also resulted in the development of parents' adaptation and mental calmness. Furthermore, other family members feel less mental suffering and tiredness so that their efforts change into satisfaction being backed by belief in God and spiritual rewards. In line with this study, in another study, spirituality was described as a stronger and more dynamic source of support than organized religion in coping with stressors and life's challenges associated with raising a child with Down syndrome [53].

The findings of this study showed the role of spirituality and religious beliefs in the acceptance of DS children. The results of the study by Bryant et al. (2011) in Pakistan also showed that “knowing the involvement of God's will” in the creation of people with Down syndrome played an important role in their acceptance by the family and parents [50]. Many other studies also showed that acts of worship, especially in Muslims, have a significant ability to adapt to adversity develop mental-psychological peace reduce stress and anxiety [54, 55], improve the quality of life [56], and develop. It has given hope and meaningful life to patients and their families [57, 58].

“Moral teaching of child” was another main theme emerging from the experiences of the participants in the present research. In fact, after searching for and developing the information and support resources of the parents and accepting the child in the light of the development of these information-support resources and the existence of religious beliefs, the family and especially the parents tried to maintain and develop the child's physical health and moral teaching and put the growing child's behavior at the forefront of their efforts.

The teaching of moral principles to children was done through different behavioral ways by family members, especially parents. Previous studies have shown that DS children receive insufficient moral teaching due to cognitive and mental problems and they are retarded in this aspect compared to other children [59].

Despite this, many studies have not been published on the issue of the moral teaching of DS children and the experiences of parents in this field, but in the study by Phillips et al., families used less authoritative, argumentative, and verbal violence and as compared to other mothers, they had more easy-going parenting methods and applied more indifference to the child's misbehavior. However, these methods have increased the level of concern of parents regarding their children's behavioral and moral qualifications [60]. Although the parents of this study also used methods such as neglect in order not to reinforce the children's misbehavior, based on the data, the participants had more supervision and sensitivity in the field of moral and behavioral teaching of their children. They not only used verbal teaching but also tried to provide suitable behavioral and moral models for their children both in their usual behavior and by using methods such as games and showing others how to deal with this. Children interact so that they have proper moral and behavioral performance while showing proper social behavior.

“Helping child's relative self-support” was also one of the main themes emerging from the experiences of the participants in the recent study. While the efforts of the parents for the moral upbringing of the child paved the way for him to be present and accepted in the community, the parents and other members of the child's family also tried to raise the DS child in such a way that his developmental skills were developed and despite mental limitations, he has gained the most individual independence in doing his personal affairs so that he has the least dependence on others in the future. In line with this study, Muñoz-Llerena acknowledged that well-defined and structured physical activity programs allow for improvements in physical, psychological, emotional, and social well-being, greater motor control, greater autonomy and independence, and performance in functional activities of daily life, avoiding the isolation of people with DS and including them in society [61].

Giving these children a chance to do their own personal affairs gradually, asking their opinions with regard to personal and familial affairs, and using special consultation and services in terms of rehabilitation, skill-learning, and speech therapy including alternative ways used by parents and family are several alternatives for making DS children independent gradually and developing their empowerment.

Rassafiani et al. believed that one of the signs of accepting a DS child in the family is that they would be treated like a typical development child [44]. Therefore, the family's struggle to make the children independent considers this as a belief in their personal and humane qualifications. The findings of this study are in line with the results of Paster et al. [46]. They found that a family with a disabled child is trying to use planned problem-solving methods in educating children with DS [46]. Self-supporting of DS children does not only decrease parents' concern about parents and family care burden [39], but also, as Alaee et al. reported, it reduces parents' concerns about the future [62]. Rassafiani et al. believed that early parents' actions in developing dependency for DS children in addition to developing self-support skills, sensory-motor skills, perceptual-motor, and also emotional skills will lead to their independence in adulthood [44].

Participants in this study have emphasized the role of children's independence in the improvement of their life quality and also their mental and physical health. In recent studies, families have tried to make these children independent so that they can afford to do their affairs even after their parents have died. Pour Mohammad Reza et al. believed that parents' early intervention would have led to DS children's independence in adulthood. Early interventions in the development of learning self-help, sensory-motor, and motor-perceptual skills can result in improvement of emotional relations between the neonate and the mother and also other persons. All these actions will direct children to gain more personal independence [63].

Also, Alaee et al. showed that parents had serious concerns about their children, but their concerns decreased with the improvement of the recovery process and the quality of life of the child. Parents who felt that they had succeeded in developing children's capabilities and their independence were less concerned in this respect [62]. Long-term dependency of children on their parents for fulfilling their needs leads to make different roles for parents. In addition, they face families with more challenges in responding to and managing care, especially, for mothers as the main members of providing their children with care needs. These make them unable to take care of their children, themselves, and other family members [39].

In this study, the presence of a disabled child in the family caused to empathize family to help the child. A child's moral teaching and making him/her familiar with rituals and the process of actions interaction with others can bring about independence for them in the future. Rassafiani et al. believed that changes in children's behaviours will result in improving their daily interactions in the family. Therefore, teaching activities accompanied by behavioral management strategies can be completely effective when skills and knowledge transmission are done by the family [63].

The next alternative being used by families to provide self-support was “developing familial support” to support the main caregivers and help them take care of the DS child. This theme had two subthemes including “strengthening the support and participation in the family” and “following up to receive support services.”

The first mentioned subtheme represents that participants came to the conclusion that looking after a DS child depends on family member's participation in the care process in addition to showing their affection and sympathy towards the main caregiver in order to support him/her in this hard pathway and decreases mental and physical pressure which they endure. Ahmadloo et al. acknowledged that the family plays an important role in the lives of DS children regardless of whether the child with intellectual disability lives at home or in support institutions. The relationship between them, family, and social interaction is a critical issue that needs to be considered [64]. Also, Van der Veek et al. stated that a DS child requires patience, being alive, growing up, and even having exposure to constant challenges. Therefore, his/her social skills should be improved [65]. The findings of Cross et al. also reported that both African American and Black Caribbean adolescents provide and receive a substantial amount of support from their families [66]. Also, the results of another study showed the value and importance that mothers place on their children's autonomy, as well as the specific factors that influence the support they provide [27].

Another theme from this study is the development of child's social interactions, which includes three subthemes of teaching social interactions to a child, prevention of social isolation, and encouraging him/her to communicate with others. In line with this study, Claire et al. demonstrated that people with Down syndrome may experience low self-esteem that may be linked to communication. There is general agreement that there is a link between self-esteem and various social and emotional difficulties. It could be argued that avoidance of situations may also have additional effects such as reduced confidence and increased mental health concerns. This highlights some of the potential effects of communication difficulties on mental health [67]. Also, a few seminal studies have demonstrated that those with a disability may be at a higher risk to be excluded when compared to TD peers [9]. On the other hand, it has been shown that lack of social support, and the widespread stigmatization confronting children with DS and their families, hinder the development of positive and empowering adjustments that would best serve the child's and the family's interests [10]. Some conducted studies have demonstrated that parents with DS children have guilt feelings, loneliness, hopelessness, grief, anger, and shame. Care burden, in addition to their social and familial roles which they have, also can lead them to fail to provide any care that the DS child, other family members, and parents require. But, if their received support is increased, their negative feelings will be lessened [38, 39]. Regarding recent findings, King et al. found that appreciation for the hard work done by the caregivers of DS children and supporting them more lead to increased integration feeling, hopefulness in the future, meaningfulness in life, and achieving power to face difficulties in families. These resulted in more adaptation with hardiness [68].

In this study, family members supported each other intimately. They tried to strengthen supportive ties by sympathizing with parents, helping each other, especially the DS child, and showing affection to other family members and the DS child. They also made all efforts to prevent any of their family members from mental and emotional traumas or, at least, diminish them. In line with this study, Roll and Bowers declared that the building approach includes strategies that rely on family members and close friends to build a support network for the person with DS. The connecting approach includes strategies that connect the person with DS to external and often professional resources and services [12].

Shakoor et al. believed that a disabled child in a family may lead to emotional agitation, endurance reduction, a tendency to escape from home, and other important changes in all aspects of family life [69]. In the same direction, Næss et al. reported that children with DS had more social problems than the typically developing controls with a similar chronological age and those with a similar nonverbal mental age [25]. In spite of previous studies where the negative results of having a disabled child in the family, prevented the family and the child from communicating with the community, in the recent study, the family members of the Down syndrome child tried to be more understanding and empathetic and showed love, respect, and empathy to the child and reduced the negative effects caused by the presence of this child in the family. Samadi et al. conducted a study in order to investigate the impacts of autistic children on a family's stress level, family function, family emotional health, satisfaction of taking roles, and informal support among Iranian parents. The findings have shown that the function of families with autistic children was weaker and parents had higher levels of stress. It was revealed that they experienced less emotional health and the mothers' stress level was higher in single-parent families and those families whose children had behavioral problems. The presence of relatives in a family and other informal supportive sources increases parents' emotional welfare, but it does not affect family stress and function. In addition, the parents with higher satisfaction of caregiving had lower stress and more emotional health [48]. The findings of McConnell et al. also showed that families with disabled children and behavioral problems and with high levels of social support had little financial pressure compared with families with low levels of social support, which had many financial problems. The research findings confirmed that flexibility in the family is more related to the availability of social and cultural support resources than individual, internal, or family factors in the family [70].

The results of these studies have revealed that emotional and social support can lessen the negative impacts of the presence of a disabled child in a parent's life. This support can originate from family members, relatives, people in society, and informal and formal sources. Choe and Hung studied the factors affecting mother's quality of life for disabled children. They also found that social support affects life quality through pressure and stress induced by parenting [49]. Also, the results of Slayter showed that families' willingness to entrust their disabled children to care centers has increased. Of course, some people also take these children from the care centers and adopt them, but in many cases, these children are returned to the care centers because of parental neglect physical and sexual abuse or lack of proper shelter to keep them. In any case, both these children who are kept in care centers and those who are adopted and returned suffer from injuries and disorders caused by being away from the family and the natural pattern of life, which endangers the child's mental health [52].

In Iran, leaving children and taking them to care centers occurs scarcely due to social, cultural, and religious conditions. In other words, families take care of their DS children themselves despite all the problems they face and the high financial pressure related to caregiving. Also, adoption is not as acceptable as what it is seen in other nations.

## 4. Conclusion

Based on the experiences of participants in the present study, although the endurance of care burden in the growth and development processes of DS children is so considerable, families make all their efforts to be adapted to this condition and also they can provide suitable circumstances for their children's education and development. The main alternatives of families to self-supporting DS children included “trying to find information-support resources,” “giving importance to child's health,” “religious beliefs of the family,” “child moral education, helping to child's relative self-support,” “developing familial support,” and “developing child's social interactions.”

These alternatives assist families to endure mental problems more efficiently in addition to being adapted to their conditions. They can also be helped how to educate their children independently and interact with others socially. Although several centers and organizations provide different services for their children, their experiences have shown that they are not enough with respect to quantity and quality.

Also, empathy of families, friends, and relatives and, especially, the understanding shown by people in the community can be very helpful in decreasing families' and DS children's pain and suffering. Culturalization being done by national broadcasting agencies can increase public awareness about these children and foster their life quality and their family members. In addition, supporting measures being taken by governmental and nongovernmental agencies can be useful as well. The findings of this study can be used by trustees of DS children's affairs and DS children's families to provide supportive care for their DS children.

## Data Availability

Data are available on written request to the corresponding author.
